# Angiopoietin-2 Is Critical for Cytokine-Induced Vascular Leakage

**DOI:** 10.1371/journal.pone.0070459

**Published:** 2013-08-05

**Authors:** Andrew V. Benest, Karoline Kruse, Soniya Savant, Markus Thomas, Anna M. Laib, Elias K. Loos, Ulrike Fiedler, Hellmut G. Augustin

**Affiliations:** 1 Division of Vascular Oncology and Metastasis, German Cancer Research Center (DKFZ-ZMBH Alliance), Heidelberg, Germany; 2 Department of Vascular Biology and Angiogenesis Research, Medical Faculty Mannheim (CBTM), Heidelberg University, Heidelberg, Germany; 3 ProQinase GmbH, Freiburg, Germany; 4 German Cancer Consortium, Heidelberg, Germany; Beth Israel Deaconess Medical Center, United States of America

## Abstract

Genetic experiments (loss-of-function and gain-of-function) have established the role of Angiopoietin/Tie ligand/receptor tyrosine kinase system as a regulator of vessel maturation and quiescence. Angiopoietin-2 (Ang-2) acts on Tie2-expressing resting endothelial cells as an antagonistic ligand to negatively interfere with the vessel stabilizing effects of constitutive Ang-1/Tie-2 signaling. Ang-2 thereby controls the vascular response to inflammation-inducing as well as angiogenesis-inducing cytokines. This study was aimed at assessing the role of Ang-2 as an autocrine (i.e. endothelial-derived) regulator of rapid vascular responses (within minutes) caused by permeability-inducing agents. Employing two independent *in vivo* assays to quantitatively assess vascular leakage (tracheal microsphere assay, 1–5 min and Miles assay, 20 min), the immediate vascular response to histamine, bradykinin and VEGF was analyzed in Ang-2-deficient (Ang-2^−/−^) mice. In comparison to the wild type control mice, the Ang2^−/−^ mice demonstrated a significantly attenuated response. The Ang-2^−/−^ phenotype was rescued by systemic administration (paracrine) of an adenovirus encoding Ang-2. Furthermore, cytokine-induced intracellular calcium influx was impaired in Ang-2^−/−^ endothelioma cells, consistent with reduced phospholipase activation *in vivo*. Additionally, recombinant human Ang-2 (rhAng-2) alone was unable to induce vascular leakage. In summary, we report here in a definite genetic setting that Ang-2 is critical for multiple vascular permeability-inducing cytokines.

## Introduction

A combination of sprouting angiogenesis-initiating cytokines, vascular guidance molecules and regulators of vessel maturation coordinate the growth of new blood vessels. The Angiopoietins (Angiopoietin-1 [Ang-1] and -2 [Ang-2]) have been identified as agonistic and antagonistic ligands, respectively, of the vascular-specific receptor tyrosine kinase Tie-2 controlling multiple vessel maturation-related signaling pathways [1], [2], [3]. Recent work suggests that Ang-2 acts as context-specific agonistic and antagonistic ligand of Tie-2 exerting agonistic effects on angiogenically activated endothelial cells and antagonistic effects on resting Tie-2-expressing cells [4], [5], [6]. Mechanistically, Ang-2 acts as an autocrine cytokine. It is produced by the endothelium and stored in Weibel-Palade bodies from where it can be rapidly released upon stimulation [7], [8], [9]. The rapid release from endothelial cells supports the hypothesis that Ang-2 functions as dynamic modulator of endothelial Tie-2 activation that controls rapid vascular responses [10].

Loss of Ang-2 does not result in embryonic lethality [11] unlike the loss of Ang-1 or Tie-2 [3], [12], [13] or the transgenic overexpression of Ang-2 [2]. Furthermore, loss of Ang-1 or Tie-2 essentially phenocopies Ang-2 overexpression supporting an antagonistic mode of action concept. Ang-2^−/−^ mice display only minor disturbances in developmental vascular morphology, but neonatal mice die by postnatal day 14 (95%–99%) due to severe lymphatic defects [11]. However, the phenotype of Ang-2^−/−^ mice is dependent on the genetic background; Ang-2^−/−^ C129J mice die postnatally, whereas Ang-2^−/−^ mice backcrossed onto a C57/Bl6 background develop to adulthood and show reduced phenotypic abnormalities [9].

Functional experiments in Ang-2^−/−^ mice have established Ang-2 as a sensitizer of inflammation- and angiogenesis-inducing cytokines [9]. Based on the cytokine-induced release of Ang-2 from activated endothelial cells and the established anti-permeability effects of paracrine Ang-1, we hypothesized that autocrine Ang-2 may similarly act as sensitizer to very rapidly acting vascular leakage-inducing cytokines. To this end, we used two different *in vivo* models to quantify vascular leakage in Ang2^−/−^ mice when exposed to different leakage-inducing factors. These experiments identified Ang-2 as essential regulator of multiple vascular leakage-inducing cytokines affecting PLCγ activation and intracellular calcium influx in response to vasoactive cytokines.

## Materials and Methods

### Reagents

All materials and reagents were purchased from Sigma Aldrich unless otherwise stated.

### In vivo procedures

All experiments were performed using 8 to 12 week-old female mice. Ang-2^−/−^ mice were crossbred into a C57/Bl6 background [9] (Ang-2^−/−^ mice originally generated as 129/J mice were kindly provided by Regeneron Inc, Tarrytown, NY, as previously described [11]). All experiments were approved by the Regierungspräsidium Karlsruhe (license no.: 35-9185.81/G-15/07) and performed according to governmental and institutional guidelines. Mice were anesthetized by intraperitoneal injection of ketamine (87 mg/kg) (Pfizer, Berlin, Germany) and xylazine (13 mg/kg) (Bayer, Berlin, Germany). For adenoviral-mediated rescue experiments, mice received an intravenous injection of 10^9^ plaque-forming units (PFU) per mouse of Ang-2 (N- terminal myc-tagged) [14] or lacZ expressing adenovirus. Blood was taken 3 days after adenoviral inoculation and serum concentrations of Ang-2 were determined by semi-quantitative Western blot using known concentrations of rhAng-2 standard. Briefly, myc-tagged hAng-2 was immunoprecipitated from the serum; 50 µl serum was precleared using protein G sepharose beads (GE Healthcare, Munich, Germany) for 2 h at 4°C followed by overnight incubation with 30 µl protein G sepharose and 3 µg mouse monoclonal anti-myc antibody (clone 9E10, Santa Cruz Biotechnology, Santa Cruz, USA).

### Measurement of tracheal microsphere extravasation

Sites of vascular leakage in tracheal blood vessels were marked by extravasated 100 nm diameter fluorescent polymer microspheres (20 µl of slurry, Duke scientific, Palo Alto, CA). Mice were anesthetized with ketamine and xylazine. Mice received intravenous injection of microspheres (20 µl) and cytokine (dissolved in saline or CHAPS) into a total volume of approximately 170 µl into the tail vein. Saline or CHAPS were used as negative control. Excess intravascular microspheres were removed from the bloodstream at designated time points by vascular perfusion of 1% paraformaldehyde (PFA) in phosphate buffered saline (PBS) (20 min, 1.5 ml/min). Mice were sacrificed and tracheas were removed and fixed for an additional 2 h in 4% PFA. Tracheal whole mounts were excised and washed twice with PBS-plus (PBS+0.3% TritonX100), blocked for 1 h in PBS-plus/1% BSA/5% goat serum and incubated overnight with the first antibody (hamster anti-mouse CD31, clone 2H8, Chemicon, Temecula, CA). Subsequently, tracheas were washed 5 times with PBS-plus and incubated for 4 h at room temperature with Texas Red conjugated goat anti-rat IgG (Jackson ImmunoResearch). Finally, tracheal whole mounts were washed 5 times, trimmed, flattened and mounted in fluorescent medium (DAKO Germany GmbH, Hamburg).

To quantify extravasated microspheres, 35 to 40 pictures were obtained from each tracheal whole mount using fixed camera settings (10× objective, IX50, Olympus). Area density of extravasated microspheres (expressed as percentage) was calculated using CellF (Olympus, Hamburg, Germany) and determined as the proportion of pixels having fluorescence intensity equal to or greater than the threshold value (default setting from 50 nm pixel size). All data are presented as mean microsphere area density ± standard deviation. Each projection represents a stack of 50 to 60 optical sections, each 0.15 µm in thickness.

### Histamine

To assess concentration-dependent vascular leakage, histamine (0.1, 0.5, 2.5, 12.5, 62.5, 312.5 µg/g) was injected intravenously followed by 20 µl microspheres diluted in 150 µl saline. Mice were perfused 90 s later with 1% PFA. To measure time-induced vascular leakage, mice received a bolus of 62.5 µg/g histamine diluted in 100 µl saline that was injected within 10 s into the tail vein. At defined time points (0, 1, 2, 3, 5, and 10 min), mice received an intravenous injection of 20 µl microspheres diluted in 150 µl saline (lag time of up to 10 s). Subsequently, mice were tail-vein perfused with 1% PFA to remove circulating microspheres and begin the fixation process.

### VEGF, Bradykinin

VEGF (0.25 µg/g) and Bradykinin (100 µg/g) were injected into the tail vein with 20 µl microspheres diluted in 150 µl saline. Mice were perfusion fixed with 1%PFA after 15 min.

### Angiopoietin-2

Recombinant Ang-2 was purified as described [9] and its biological activity was confirmed by *in vitro* sprouting [15]. Additionally, commercially available recombinant Ang-2 (R&D Systems GmbH, Wiesbaden, Germany) was used, yielding essentially the same results. Following intravenous injection of 0.1, 0.5 and 1 µg/g Ang-2 together with 20 µl microspheres diluted in 150 µl saline (60 min), the remaining microspheres were flushed from the bloodstream by perfusion of 1%PFA. CHAPS was used as a negative control.

### Miles assay

To measure albumin-bound Evans Blue dye leakage from the vasculature into the interstitium, the ventral fur of the mouse was removed with depilatory cream. Two days later, mice received an intravenous injection of Evans Blue dye (1% in PBS). Mice were anesthetized with isoflurane and injected intradermally with cytokines (histamine 25 ng, 125 ng or 625 ng, VEGF 25 ng and bradykinin 100 ng) diluted in 20 µl saline. Saline was used as negative control. 20 min later, mice were sacrificed, the tissue containing extravasated dye was removed and incubated overnight in 200 µl formamide at 55°C. Extracted Evans Blue dye was quantified using a spectrophotometer set at 650 nm wavelength and expressed as mean ± standard deviation normalized to tissue weight.

### Electron microscopy

Tissues for electron microscopy were prepared as previously described [16]. Mice were sacrificed by carbon dioxide asphyxiation to prevent damage to the tracheal tissue.

### Generation of endothelioma cells

Mouse lungs were isolated from wild type and Ang2^−/−^ mice. Lungs were digested in an equal volume of Collagenase (Collagenase II, Biochrom) for 30 min at 37°C, with regular shaking. Tissue was washed twice in HBSS (Gibco)/10% FCS and pelleted at 1100 rpm for 10 min before resuspension in HBSS/10% FCS and passed through a 40 µm cell strainer. The CD31-Dynabead conjugate (per lung pair; 30 µL sheep anti-rat IgG Dynabead+20 µL rat-anti mouse CD31 (MEC13.3, BD Pharmingen), which was incubated overnight at 4°C in 950 µL HBSS/0.1% BSA) was then added for 30 min at 4°C before magnetic selection as per manufacturer's instructions. Murine lung endothelial cells were plated onto cell culture plates coated with 1% gelatin (Bovine skin, type B) and cultured at 37°C in DMEM (high glucose, with GlutaMAX, sodium pyruvate; Gibco, Invitrogen) supplemented with 10% FCS, 1% penicillin/streptomycin, 1% non-essential amino acids, and 5×10^−5^ 2-mercaptoethanol. Endothelial cell growth supplement (Sigma-Aldrich) was added as a source of growth factors. Endothelioma cell lines from Ang2^−/−^ mice were established the following day by infection with a recombinant retrovirus transducing the polyoma virus middle T oncogene as previously described [17]. Expression of CD31 was confirmed by immunofluorescence.

### Endothelial cell culture

Human umbilical vein endothelial cells (HUVEC, Promocell, Heidelberg, Germany) were cultured in endothelial cell growth media supplemented with 10% fetal calf serum (FCS) purchased from Promocell (Heidelberg, Germany). HUVEC were serum starved overnight in endothelial cell basal medium supplemented with 2% FCS and experiments were performed in endothelial basal medium without FCS.

### Protein extraction and Western blot analysis

Mice were treated with histamine or saline for 5 min as previously described and then sacrificed. Lungs were excised and briefly washed in ice cold PBS containing 2 mM sodium orthovanadate. They were lysed in ice cold lysis buffer containing 2 mM sodium orthovanadate, 50 mM NaCl, 50 mM Tris-HCl, pH 7.4, 1% NP-40, 10 mM EDTA, 10% Glycerin, 1% Protease inhibitor cocktail (Mix G, Serva) and manually homogenized on ice. Samples were further lysed for 1 h at 4°C on a rotator, or centrifuged and clarified lysates were then boiled in standard SDS containing sample buffer for 10 min. Samples were immunoprecipitated using antibodies to Tie-2 (Clone 4G8 (Chemicon International, Maryland, USA), PLCγ2 (#3872, New England Biolabs, Frankfurt, Germany) and Western blots were probed with antibodies to Tie-2, phosphotyrosine (4G10, Chemicon International, Maryland, USA), PLCγ2, phospho-PLCγ2 (#3871, New England Biolabs, Frankfurt, Germany). Actin was probed using the antibody SC-1616 from Santa Cruz. Autoradiographic films were developed, digitally scanned and converted to 8-bit images. The gel intensity analysis was performed using ImageJ and following background reduction, the integrated intensity was quantified and compared using a Students t-test.

### In vitro cellular calcium quantification

HUVEC as well as transformed lung endothelial cells (EOMA) from wild type (WT) and from Ang-2^−/−^ mice (KO) were cultured in 96-well assay plates. Growth medium was removed and cells were loaded with a Fluo-4 dye solution (Fluo-4 NW Calcium Assay Kit, Invitrogen, Karlsruhe, Germany). Assay plates were incubated at 37°C for 30 min and then at room temperature for 30 min. Cells were then stimulated for 10 min with either VEGF (25 ng/ml) or histamine (10^−5^ M) and fluorescence was measured using a microplate reader (Infinite 200, Tecan Group Ltd., Germany) with excitation at 494 nm and emission at 516 nm.

### Statistical analysis

Statistical analyses were performed using one-way ANOVA with post-hoc Bonferroni or Student's t-test where appropriate. P values of less than 0.05 were considered statistically significant. All data are presented as mean ± standard deviation.

## Results

### Loss of Ang-2 caused tracheal blood vessel ultrastructural changes

Previous work examining the vascular ultrastructure of postnatal Ang-2^−/−^ mice has focused on the lymphatic vessels, where the most pronounced functional phenotype was observed [11], [18]. To investigate this further, we performed electron microscopy (EM) of tracheal microvessels in stimulated and unstimulated Ang-2^−/−^ (Supplementary Fig. S1A, B) and WT mice (Supplementary Fig. S1C, D). Upon histamine stimulation (approx. 11 min), no marked alterations were observed in the vessel structure. Yet, most strikingly the endothelium of Ang-2^−/−^ mice appeared thickened with a wider basement membrane.

### Loss of Ang-2 attenuates histamine-increased vascular leakage

**Figure 1:**
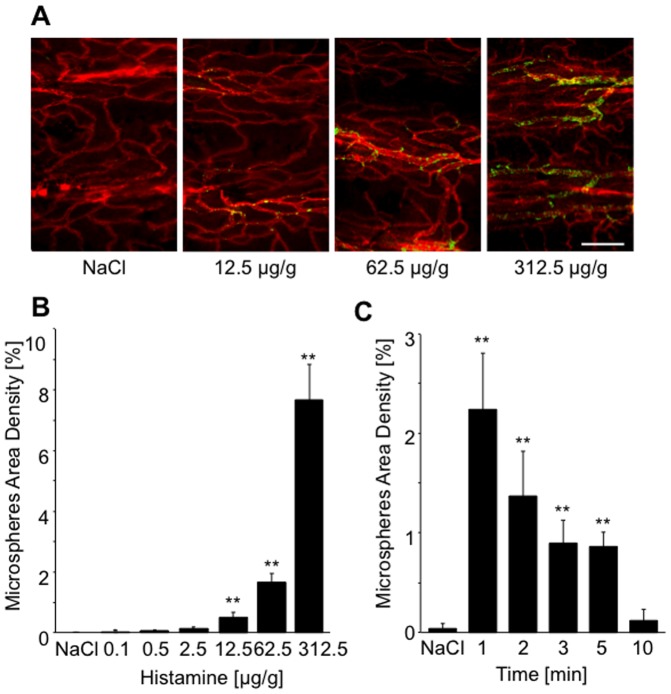
Time and dose dependency of histamine-induced vascular leakage in the tracheal microsphere extravasation assay. Mice were intravenously injected with increasing concentrations of histamine (0.1, 0.5, 2.5, 12.55, 62.5 and 312.5 µg/g) or saline as negative control together with 100 nm green fluorescent microspheres. Tissue samples were whole-mount stained for CD31 (**A**) and microspheres area density was quantified as indicated in Materials and Methods (**B, C**). To establish the dose response of histamine-induced vascular leakage, fluorescent microspheres were injected together with histamine and mice were sacrificed after 1 min (**B**). To establish the time course of histamine-induced vascular leakage, microspheres were injected after the indicated times of exposure to submaximal concentrations of histamine (62.5 µg/g) and mice were sacrificed within 1 min (**C**). Values are expressed as mean ± SD (n = 5, saline n = 3). **P<0.01 compared to saline. Scale bar 100 µm.

**Figure 2:**
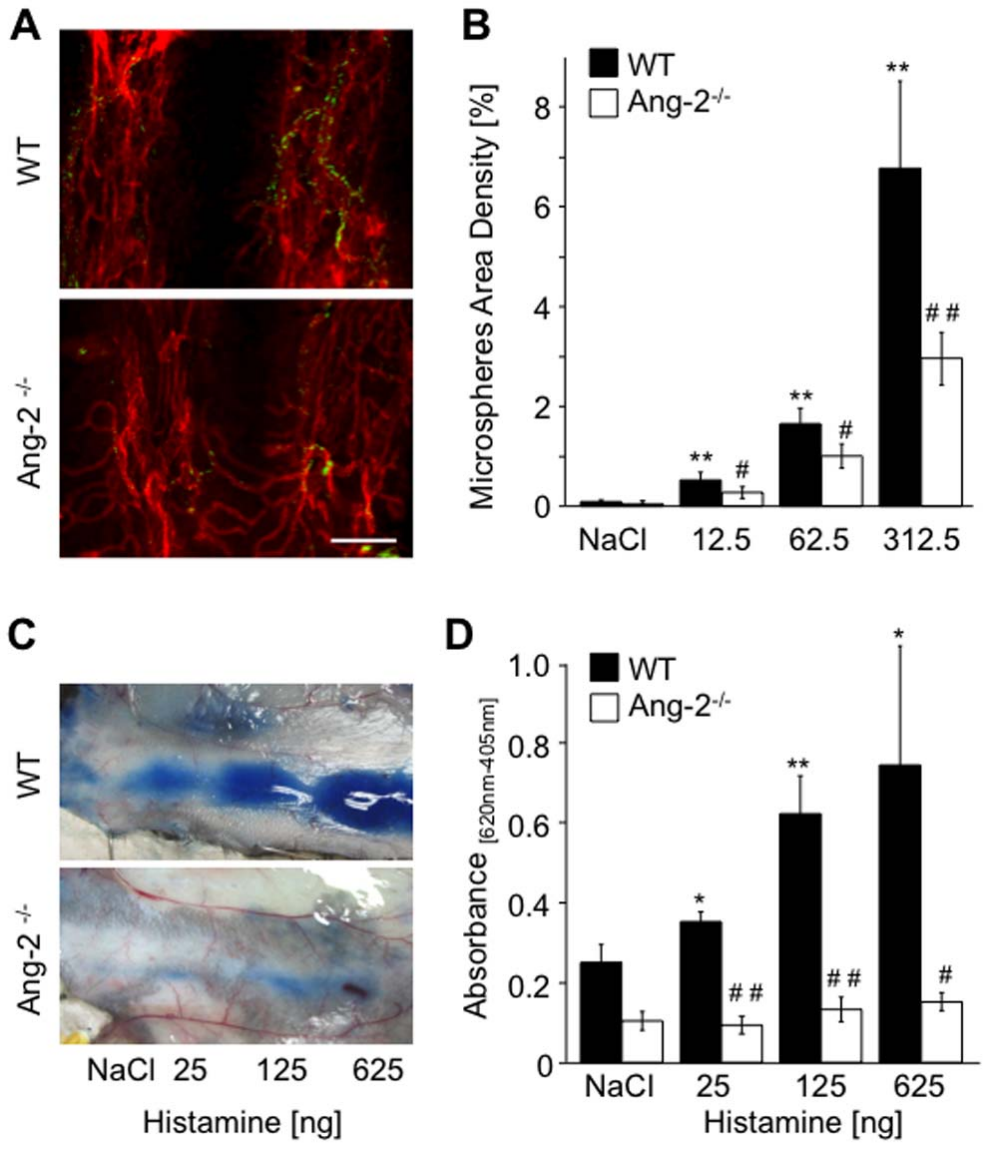
Histamine-induced vascular leakage is reduced in Ang-2^−/−^ mice. WT and Ang-2^−/−^ mice were intravenously injected with 12.5, 62.5 and 312.5 µg/g histamine or saline as negative control together with 100 nm green fluorescent microspheres. Tracheas were whole-mount stained for CD31 (red, **A**), and microspheres area density was measured (**B**). The response to locally administered histamine on vascular leakage was measured using the Miles assay. Increasing concentrations of histamine (25 ng, 125 ng, 625 ng) or saline as negative control, were injected intradermally in WT and Ang-2^−/−^ mice (**C**) and dye extravasation was quantified (**D**). Values are expressed as mean ± SD (n = 5, trachea assay; n = 4, Miles assay). *p<0.05 compared to saline; **p<0.01 compared to saline; #p<0.05 compared to corresponding WT histamine treatment group; ##p<0.01 compared to corresponding WT histamine treatment group. Scale bar 100 µm.

**Figure 3:**
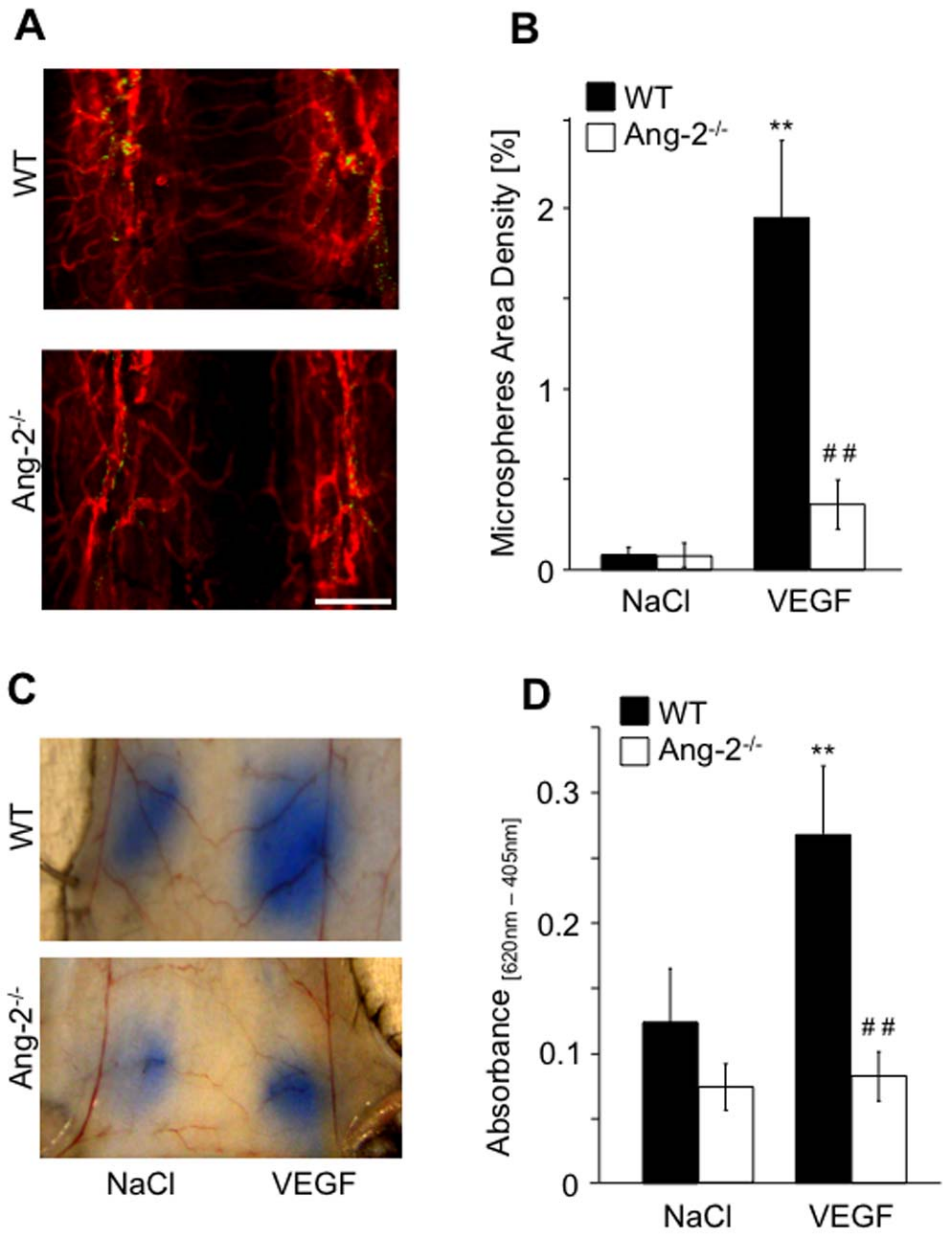
VEGF-induced vascular leakage is reduced in Ang-2^−/−^ mice. WT and Ang-2^−/−^ mice were intravenously injected with 0.25 µg/g VEGF or saline as negative control together with 100 nm green fluorescent microspheres. Tracheas were whole-mount stained for CD31 (red, **A**), and microspheres area density was measured (**B**). For Miles assays, WT and Ang-2-deficient mice were intradermally injected with 25 ng VEGF or saline as negative control (**C**). Skin patches were removed and extravasated dye was measured (**D**). Results are expressed as mean ± SD (n = 5, trachea assay; n = 3, Miles assay). **p<0.01 compared to saline; ##p<0.01 compared to WT and VEGF, respectively. Scale bar 100 µm.

**Figure 4:**
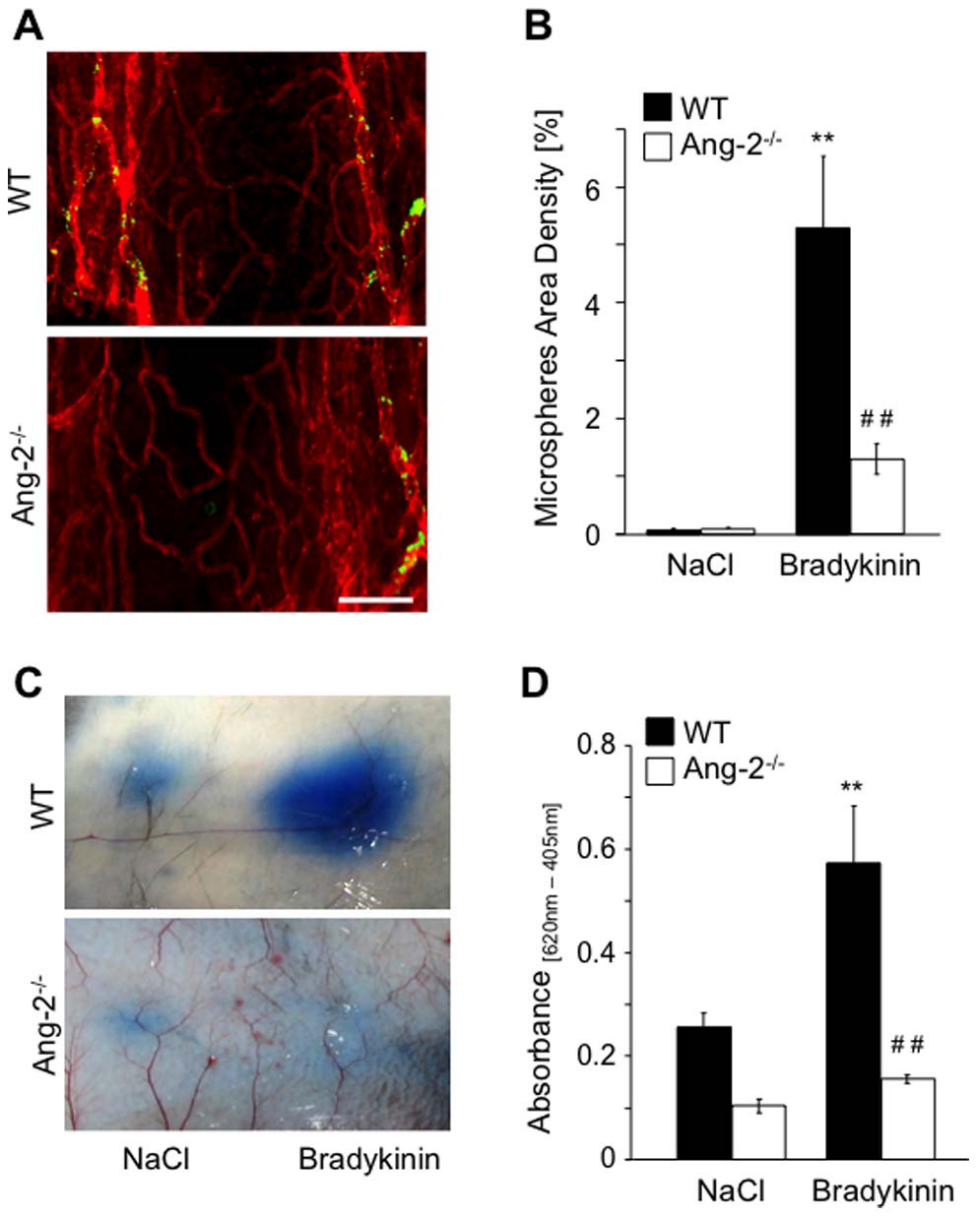
Bradykinin-induced vascular leakage is reduced in Ang-2^−/−^ mice. Bradykinin (100 µg/g) was injected intravenously together with 100 nm fluorescent microspheres in WT and Ang-2^−/−^ mice. CD31 whole-mount stained tracheas (**A**) demonstrated higher microsphere accumulation in WT mice compared to Ang-2^−/−^ mice (**B**). Miles assays was performed in WT and Ang-2^−/−^ mice. Bradykinin (100 ng) or saline as negative control were injected intradermally (**C**). Skin patches were taken and extravasated dye was measured (**D**). Results are expressed as mean ± SD (n = 5, trachea assay; n = 3, Miles assay). **p<0.01 compared to saline; ##p<0.01 compared to WT and Bradykinin, respectively. Scale bar 100 µm.

Histamine stimulation rapidly resulted in the onset of sluggish behavior in WT, but not Ang-2^−/−^ mice. A dose and time dependence protocol was carried out to establish experimental regimes for subsequent experiments to investigate the response to histamine. WT mice were treated with increasing concentrations of histamine (up to 312.5 µg/g, Fig. 1 A, B). Demonstrating a dose dependent response to histamine, maximum degree of tracheal microsphere accumulation was observed following 312.5 µg/g histamine and the microsphere density measured was 7.7% compared with 0.01% at basal levels. Additionally, a time course experiment was performed using a sub-maximal dose (62.5 µg/g) of histamine (Fig. 1C). Microsphere accumulation reached a maximum within 1 min of injection (approximately doubling basal microsphere accumulation), reduced by 3 min and returned to basal levels after 10 min (Fig. 1C). Thus, microsphere accumulation upon histamine treatment was dose- and time-dependent. An attenuated dose dependency was recorded in Ang2^−/−^ mice, and significantly reduced microsphere accumulation was recorded compared to WT responses (Fig. 2A, B, p<0.05, 12.5 µg and 62.5 µg histamine, p<0.01 312.5 µg histamine). Consistent results were obtained upon intradermal injection of histamine using the Miles assay. No increase in dye extravasation upon histamine was recorded in Ang-2^−/−^ mice compared to WT littermates (Fig. 2 C, D, p<0.01 (125 ng), p<0.05 (25, 625 ng)). Basal plasma leakage was also reduced compared to WT controls, but not significantly (Fig. 2 C, D), suggesting that Ang-2 could also play a role in regulating basal and stimulated permeability of dermal and sub-dermal microvessels (Fig. 2D, 3D, 4D).

### Loss of Ang-2 reduces VEGF-induced vascular leakage

Loss of Ang-2 resulted in a reduction in VEGF-induced vascular leakage in both vascular leakage assays (Fig. 3). WT mice demonstrated a significant increase in both microsphere accumulation in the trachea (Fig. 3A, B p<0.001) and an increase in Evans blue dye tissue concentration (Fig. 3C, D p<0.01) in response to VEGF stimulation, whereas the responses in Ang-2^−/−^ mice were significantly lower compared to control mice (p<0.01).

### Loss of Ang-2 reduces bradykinin-induced vascular leakage

Ang-2^−/−^ mice displayed a significantly reduced response to bradykinin compared to WT mice (Fig. 4). Microsphere extravasation was increased slightly in Ang-2^−/−^ following bradykinin stimulation, but this was significantly reduced compared to the effect observed in WT mice (Fig. 4A, B). Administration of bradykinin similarly led to a significant increase in Evans Blue dye accumulation in WT (Fig. 4C, D p<0.05) but not in Ang-2^−/−^ mice (Fig. 4C,D p>0.5). Moreover, baseline plasma leakage was also significantly reduced in Ang-2^−/−^ mice as they displayed a significantly reduced Evans Blue dye content following administration of saline compared to WT littermates (Fig. 4D, p<0.01).

### Angiopoietin-2 alone does not increase vascular leakage

**Figure 5:**
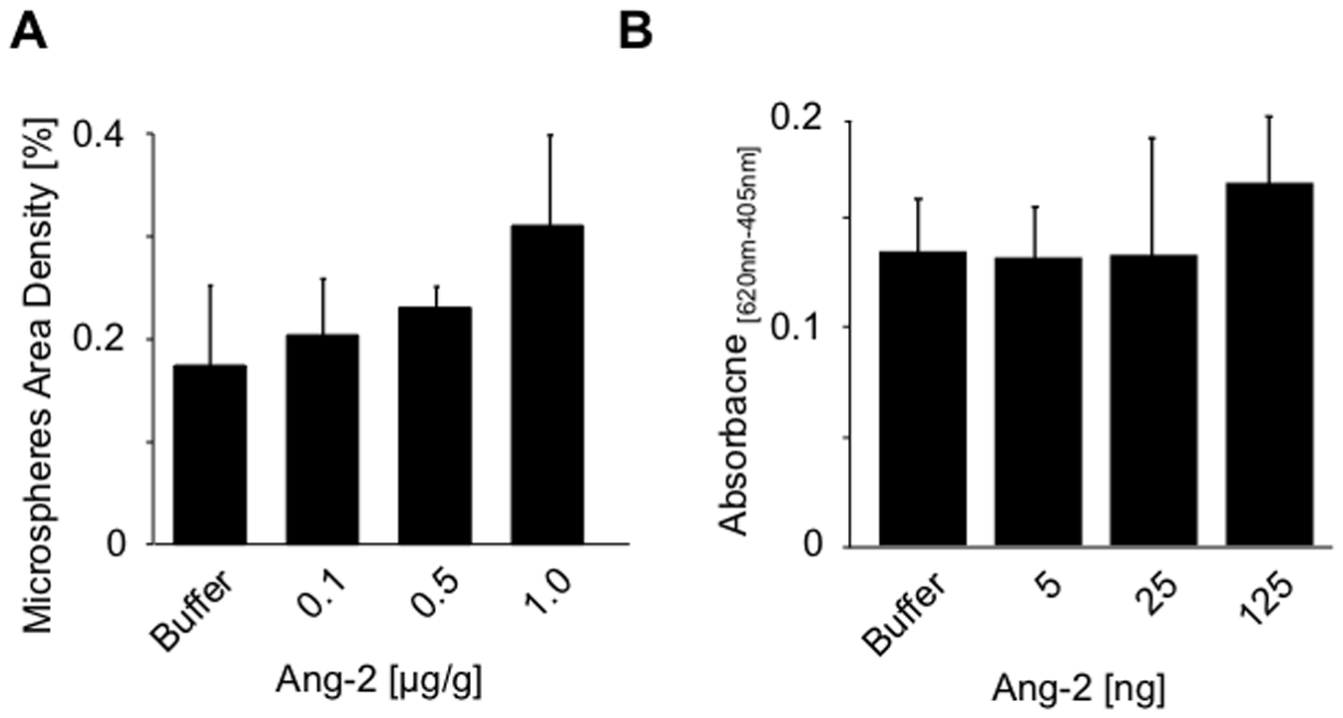
Exogenous Ang-2 does not induce vascular leakage. Increasing concentrations of recombinant Ang-2 (0.1, 0.5 and 1 µg/g) or CHAPS buffer as negative control were intravenously injected together with 100 nm green fluorescent microspheres (**A**). CD31 whole-mount stained tracheas from WT mice were quantitated for microsphere extravasation. Miles assay was performed using increasing concentrations of Ang-2 (5, 25, 125 ng), Evans blue dye was extracted from skin samples and quantified (**B**). Results are expressed as mean ± SD (n = 3, trachea assay; n = 5, Miles assay).

Bioactivity of baculovirus generated recombinant Ang-2 (rhAng-2) was confirmed by *in vitro* sprouting assay as reported previously [10] (Supplementary Fig. S2). Mice received increasing doses of rhAng-2 (0.1, 0.5 and 1 µg/g) and their tracheas were excised and stained for CD31 (Supplementary Fig. S3A). No significant accumulation or extravasation of 100 nm fluorescent microspheres was observed when compared with CHAPS buffer alone nor when compared with increasing doses of Ang-2 (p>0.05, Fig. 5A). Furthermore, Evans Blue dye leakage was also unaltered following intradermal administration of rhAng-2 (5, 25 and 125 ng; p>0.05, Fig. 5B, Supplementary Fig. S3B), establishing that Ang-2 alone does not induce significant levels of vascular leakage in the analyzed concentration range (Fig. 5B, Supplementary Fig. S3B, p>0.05).

### Ad-Ang-2 rescues the phenotype of Ang-2-deficient mice

**Figure 6:**
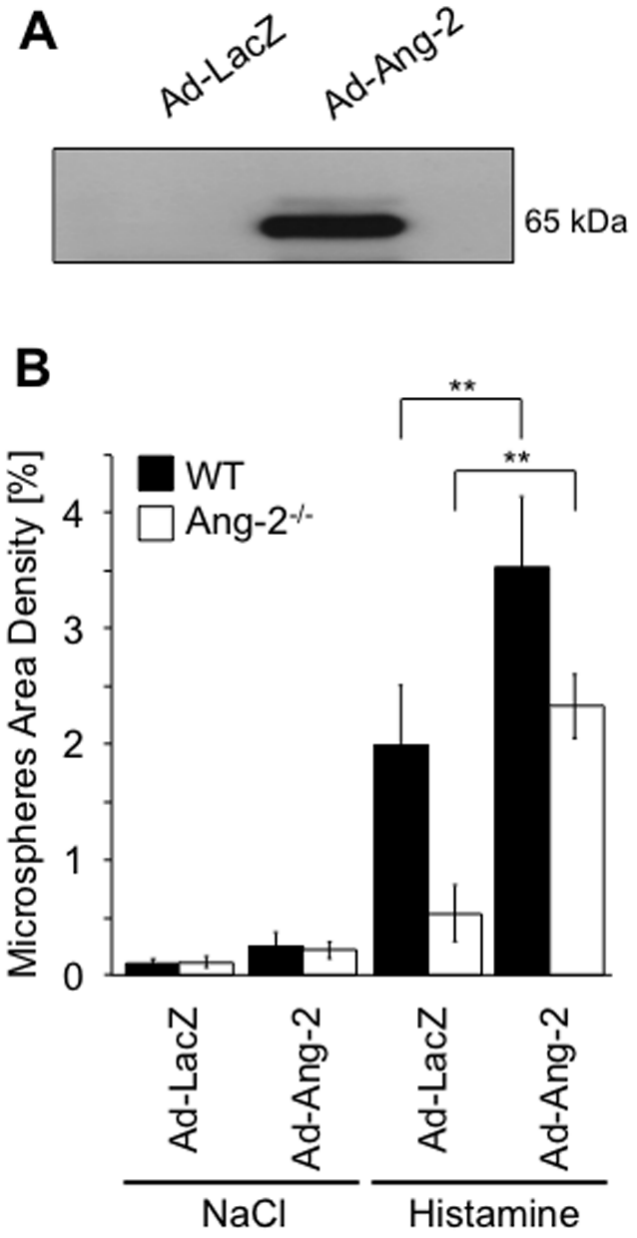
Adenoviral delivery of Ad-Ang-2 rescues the loss of histamine response in Ang-2^−/−^ mice. WT and Ang-2^−/−^ mice received an intravenous injection of 10^9^ PFU Ad-LacZ or Ad-Ang-2, respectively. Ang-2 in the serum was semi-quantitatively determined by Western blot after 3 days showing a myc-tagged band of approx. 60 kDa in Ad-Ang-2 treated mice alone (**A**). Histamine (62.5 µg/g) or saline as negative control were injected together with 100 nm green fluorescent microspheres in either Ad-LacZ or Ad-Ang-2 treated WT and Ang-2^−/−^ mice. CD31 whole-mount stained tracheas were quantitated for microsphere extravasation (**B**). Adenoviral delivery of Ang-2 in Ang-2^−/−^ mice rescued the phenotype of Ang-2^−/−^ mice and facilitated the histamine response. Moreover, delivery of Ad-Ang-2 in WT mice enhanced the response to histamine. Values are expressed as mean ± SD (n = 6, saline n = 3). **p<0.01 compared to Ad-LacZ and histamine.

Tail-vein injection of adenovirus results in transduction of the liver and subsequently long-term elevated circulating levels of adenovirus-encoded growth factor can be achieved as opposed to recombinant protein injection [9], [11]. Adenoviral treatment was well tolerated, with no observable signs of distress. The adenoviral-encoded myc tagged-Ang-2 was readily detectable in the serum following immunoprecipitation and detection using an anti-myc antibody (Fig. 6A). Neither WT nor Ang-2^−/−^ mice had any significant microsphere accumulation in the tracheal vasculature under control conditions (Fig. 6B). As suggested by others, we consider that Ang-2 acts in a paracrine manner in this experimental setting [11]. Furthermore, there was no significant difference in basal leakage following either Ad-LacZ or Ad-Ang-2 administration (Fig. 6B, p<0.01). Systemic adenoviral administration did not affect the ability of the WT trachea to respond to histamine, as both Ad-LacZ control and Ad-Ang-2 treated WT mice increased their microsphere extravasation following histamine stimulation (Fig. 6B, p<0.05 (Ad-LacZ) and p<0.01 (Ad-Ang-2). Ang-2^−/−^ mice treated with Ad-LacZ did not increase microsphere accumulation in response to 62.5 µg histamine. However, Ad-Ang-2 treatment of Ang-2^−/−^ mice resulted in an increase in microsphere accumulation compared with Ad-LacZ control (Fig. 6B, p<0.01). Moreover, histamine stimulation of Ad-Ang-2 treated WT mice led to a further increase in microsphere accumulation compared to Ad-Ang-2 treated Ang-2^−/−^ mice. Overexpression of Ang-2 in WT mice did not significantly increase microsphere accumulation compared to Ad-LacZ overexpression (Fig. 6B, p>0.05) following saline treatment. However, WT mice overexpressing Ang-2 potentiated the response to histamine, demonstrating a significant increase in microsphere accumulation compared to Ad-LacZ treated WT mice (Fig. 6B, p<0.05). Ang-2^−/−^ mice still responded to histamine but to a lesser extent than WT mice. However, the phenotype was largely rescued following Ad-Ang-2 treatment, as microsphere accumulation was significantly increased compared to Ad-LacZ (Fig. 6B, p<0.01).

### Loss of endothelial Ang-2 results in reduced ability to increase intracellular calcium

**Figure 7:**
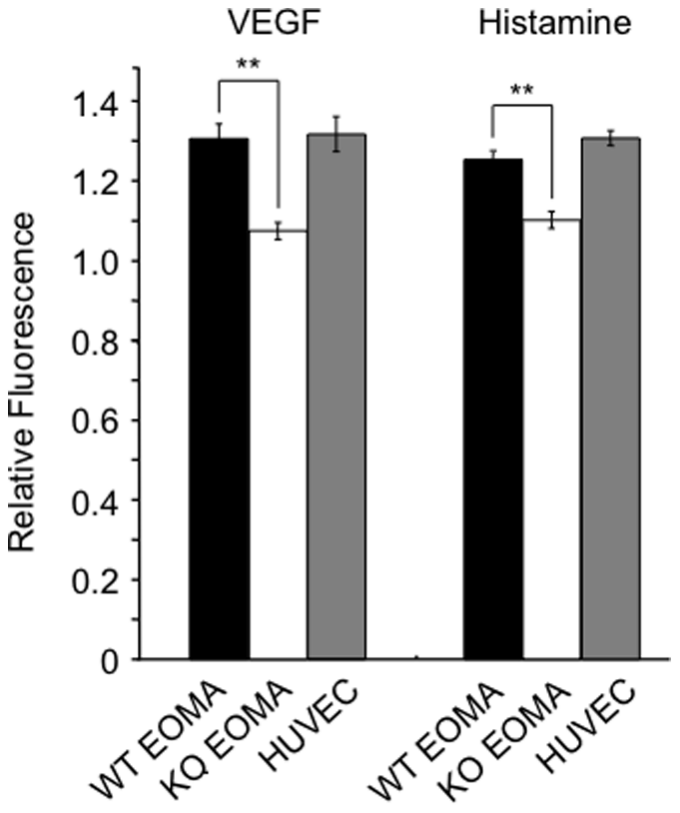
Loss of Ang-2 results in attenuated intracellular calcium increase in response to VEGF and histamine *in vitro*. Immortalized murine lung endothelial cells from WT and Ang-2^−/−^ mice (WT EOMA, KO EOMA) were loaded with Fluo-4 calcium sensitive dye and the relative fluorescence (stimulated/unstimulated fluorescence) was quantified in response to VEGF (25 ng/ml) and histamine (10^−5^M) after 10 min stimulation. HUVEC served as positive control (n = 3, **p<0.005 WT vs. KO).

Given that increased intracellular calcium is a key intracellular mediator of many permeability-increasing cytokines [19], we hypothesized that the loss of vascular leakage observed in the absence of Ang-2 could result from altered calcium signaling in endothelial cells. To this end, we used a Fluo-4-based calcium assay and demonstrated that transformed endothelial cells from WT mice (endothelioma cells) stimulated with VEGF or histamine increase intracellular calcium after 10 min of stimulation. HUVEC were used as positive control for each experiment, with both VEGF and histamine increasing intracellular calcium throughout the experiment (Fig. 7). However, Ang-2 KO endothelioma cells displayed a significantly reduced response to histamine and VEGF compared to WT control endothelioma cells (Fig. 7, p<0.005, data normalized to unstimulated). Baseline calcium levels were not significantly altered in unstimulated endothelial cells.

### Loss of Ang-2 results in reduced PLCγ2 activation

**Figure 8:**
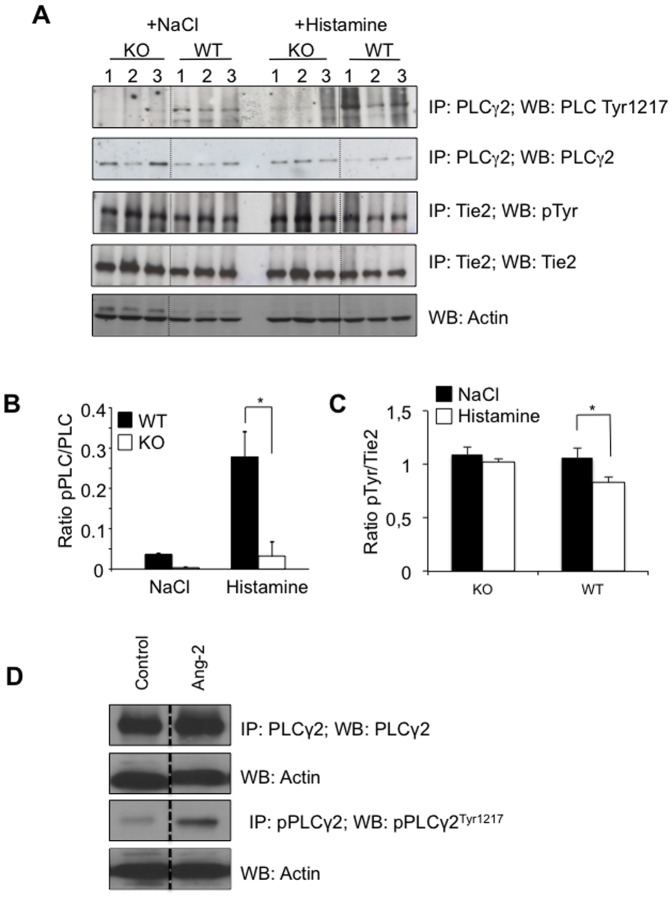
Loss of Ang-2 reduces PLCγ2 activation *in vivo*. (**A**) WT and Ang-2^−/−^ mice were intravenously injected with 62.5 µg/g histamine or saline as negative control. Mice were sacrificed after 90 s. The lungs were excised (1,2,3 represent different mice), and tissue lysates were used for immunoprecipitation and Western blotting. The blots were probed for PLCγ2, phospho-PLCγ2 (Tyr 1217), Tie-2, phospho-tyrosine and actin as indicated. Quantification of the relative intensity of phospho-PLCγ2 to total PLCγ2 (**B**) and phospho-tyrosine to total Tie-2 (**C**) revealed a significant reduction in PLCγ2 phosphorylation following histamine stimulation in Ang-2^−/−^ mice compared to WT mice and no reduction in pTie-2 following histamine in KO mice. (n = 3 animals for each group. *p<0.05). Additionally, HUVEC stimulated with recombinant Ang-2 demonstrated an increase in activation of PLCγ2 compared to unstimulated cells indicating the role of Ang-2 in PLCγ2 activation thereby affecting intracellular calcium influx (**D**).

From a range of potential effectors of calcium signaling, we demonstrated that histamine increased the activation of PLC2γ at Tyrosine1217 (Fig. 8A, B, p<0.05) in WT mice. However, the loss of Ang-2 resulted in a loss of this response, with control and stimulation levels being approximately equal. It is worth noting that PLC2γ levels were not altered in lung lysates suggesting that the difference was due to activation differences rather than total differences. Additionally, we demonstrated that during histamine stimulation, Ang-2 acted antagonistically as indicated by a reduction in phospho-Tie-2 following histamine stimulation in WT mice (p<0.05) but not in Ang-2^−/−^ littermates (p = 0.08, Fig. 8A, C). These data also show that Tie-2 phosphorylation was not blocked in Ang-2^−/−^ mice. Further validating this finding, paracrine Ang-2 stimulated HUVEC displayed increased PLCγ activation at Tyr1217 (Fig. 8D) consistent with reduced pPLCγ in Ang-2^−/−^ mice following histamine stimulation. However, this effect was not sufficient to induce increased vascular leakage as observed in the *in vivo* experiments (Fig. 5).

## Discussion

The present study was aimed at further investigating the functional consequences of loss of Ang-2 in the murine vasculature. Previously, we have demonstrated that Ang-2 can act as a sensitizer of cytokine-induced vascular destabilization [20]. Likewise, Ang-2 is essential for priming the endothelium to TNF-α-induced leukocyte adhesion in adult mice [9]. Consequently, we hypothesized that Ang-2 could also act as a sensitizing regulator of other endothelial stimuli [8]. Therefore, we sought to determine if loss of Ang-2 alters the endothelial response to cytokine-induced vascular leakage. The present study showed (i) that Ang-2 was required for the induction of vascular leakage in response to multiple vasoactive cytokines (histamine, bradykinin, VEGF), (ii) that Ang-2 did not affect vascular leakage on its own in the analyzed systemic and local concentration range (albeit being able to induce PLCγ2 in vitro), and (iii) that the sensitizing role of Ang-2 towards vascular leakage-inducing cytokines affected PLCγ2 activation and calcium influx.

Bradykinin has previously been demonstrated to rapidly induce plasma extravasation and the resulting proinflammatory effects were suppressed by systemic administration of Ang-1* (an engineered variant of Ang-1) [21]. The finding that Ang-2^−/−^ mice essentially phenocopy recombinant Ang-1 administration supports the concept that Ang-2 acts on resting Tie-2-expressing endothelial cells as an antagonist of constitutive Ang-1/Tie-2 signaling [7]. The activation of Tie-2 has previously been demonstrated to downregulate the expression of a number of pro-inflammatory cytokines [22], [23]. Yet, the functional consequences of such activation in response to different vasoactive cytokines have so far not been analyzed in great detail.

We have previously demonstrated that Ang-2 stimulation of endothelial cell/smooth muscle cell co-culture spheroids results in destabilization and subsequent loss of association between the two cell types with loosened inter-endothelial junctions, which do not involve VE-cadherin but Tie-2-integrin complexes [20], [24]. Ang-1* stimulation has also been shown to reduce inter-endothelial gaps [21] and Ang-2 transgenic mice resemble the Tie-2/Ang-1 knockout mice demonstrating a qualitatively similar phenotype [3], [12], [13]. These observations are all consistent with our finding that the loss of endothelial Ang-2 results in attenuated responses to a variety of leakage-inducing cytokines, but does not induce such effects alone. Rather, the Ang-Tie axis regulates the EC response. Cellular responses to histamine [25], [26], bradykinin [27] and VEGF all require an increase in intracellular calcium [28] and the activation of PKC and PLCγ, which results in an increase in microvascular permeability (within seconds to minutes of stimulation) [29]. Ang-1 has previously been demonstrated to alter the association of various endothelial junctional molecules (such as VE-cadherin) in response to VEGF [30], [31] or TNF-α [22]. Additionally, Ang-1 is able to inhibit VEGF induced calcium influx [32], which supports the canonical notion that activation of Tie-2 reduces intracellular endothelial calcium to reduce permeability, and consequently, antagonism of Tie-2 would likely result in the opposite. We demonstrate that the loss of Ang-2 impairs the ability of the endothelial cells to increase intracellular calcium in response to VEGF and histamine, which compliments the published evidence for the anti-inflammatory actions of Ang-1 [33], [34]. Consistent with this observation, we demonstrate reduced Tie-2 activation *in vivo* following histamine stimulation in wild type but not Ang-2^−/−^ mice, which adds further evidence that Ang-2 acts as Tie-2 antagonist on resting endothelium. In addition to altered cellular calcium handling, we hypothesized that there could be ultrastructural barriers to increase vascular leakage itself. We have demonstrated the presence of a thickened endothelium, thickened basement membrane and an additional, currently unidentified extra-membrane that could inhibit not only the passage of albumin bound Evans blue dye in the Miles assay, but also the passage of fluorescent microspheres into the interstitium. It is worth noting that administration of Ang-1 has been demonstrated to thicken the endothelial glycocalyx in frog and rat mesenteries, therefore altering solute permeability in short-term (20 min) [35] by reducing the hydraulic conductance and forming a physical barrier to large solutes. As both, the *in vivo* assays measure large solute extravasation, a thickened path length, from lumen to abluminal surfaces could result in slower leakage time, which would result in reduced accumulation of either microspheres or dye into the interstitium. Chronic stimulation of cultured EC with Ang-2 results in altered VE-cadherin signaling, and myosin light chain activation, leading to reduce EC integrity [36]. However, in this study we demonstrate a PLCγ activation mechanism as a short-term alternative to long-term mechanisms of vascular leakage which involve VE-cadherin and junctional reorganization.

In summary, Ang-2 is an essential component of cytokine-induced vascular leakage. Employing two *in vivo* assays, demonstrating both plasma leakage induced by systemic or local administration of a given cytokine, this study showed that loss of Ang-2 resulted in strong attenuation of vascular leakage following histamine, bradykinin and VEGF administration, and that this was associated with altered PLCγ activation *in vivo*. Ang-2 is presently strongly explored as target for anti-tumor therapeutic applications. The results of this study provide a strong rationale for the exploration of Ang-2 as therapeutic target in non-oncological vascular pathologies, including vascular leakage, inflammation and sepsis.

## Supporting Information

Figure S1 Ultrastructural characteristics of tracheal blood vessels in Ang-2-deficient mice. Microvessels in untreated (A) and 11 min histamine-exposed tracheal vasculature (B) with thickened endothelium (arrowhead) and a pronounced layer of basement-membrane (arrow) (insert in B: Higher power view of thickened basement membrane [arrow]). In contrast, tracheal microvessels in untreated (C) and histamine-exposed wild type mice (D) have a flattened layer of endothelium (arrowhead) with very thin basement membrane (arrow). Scale bars: 2 µm. Scale bar insert: 1 µm. (PDF)

Figure S2 Spheroid-based endothelial sprouting angiogenesis assay demonstrating the bioactivity of baculovirus-produced Ang-2. VEGF served as positive control. ** p<0.01, ***p<0.001 compared to control. (PDF)

Figure S3 Representative images of CD31-stained tracheal vasculatures (A) and Miles' Assay (B) following rhAng-2 administration. Scale bar 100 µm. (PDF)
