# Supplementary material for: Angiopoietin-2 Is Critical for Cytokine-Induced Vascular Leakage
Source: PLoS One. 2013 Aug 5;8(8):e70459. doi: 10.1371/journal.pone.0070459 (PMC3734283; doi:10.1371/journal.pone.0070459)
Supplement: Figure S1 — (PDF) [file pone.0070459.s001.pdf]

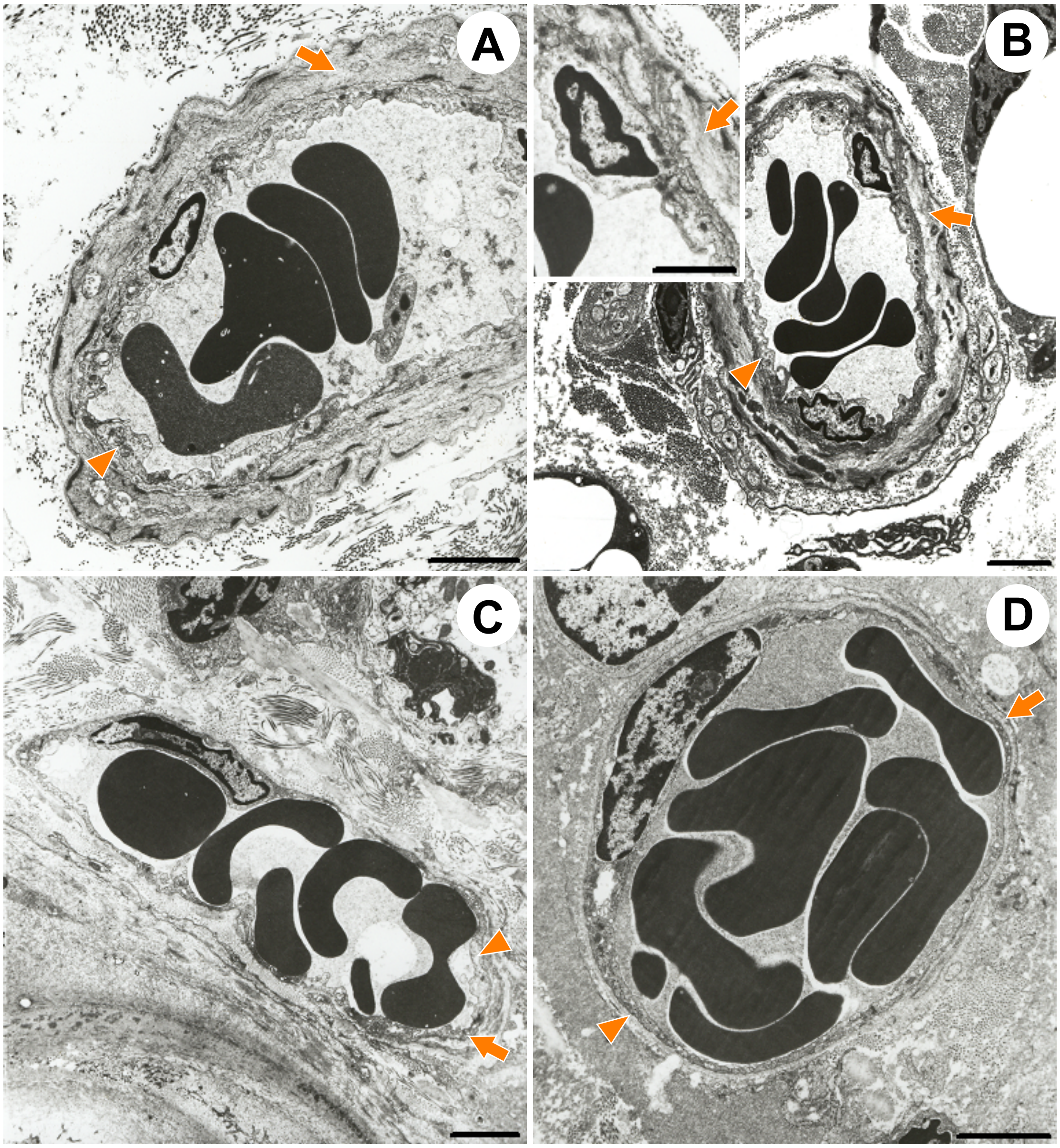

Supplementary Figure S1: Ultrastructural characteristics of tracheal blood vessels in Ang-2-deficient mice. Microvessels in untreated (**A**) and 11 min histamine-exposed tracheal vasculature (**B**) with thickened endothelium (arrowhead) and a pronounced layer of basement-membrane (arrow) (insert in **B**: Higher power view of thickened basement membrane [arrow]). In contrast, tracheal microvessels in untreated (**C**) and histamine-exposed wild type mice (**D**) have a flattened layer of endothelium (arrowhead) with very thin basement membrane (arrow). Scale bars: 2 µm. Scale bar insert: 1 µm.
