# Supplementary material for: Angiopoietin-2 Is Critical for Cytokine-Induced Vascular Leakage
Source: PLoS One. 2013 Aug 5;8(8):e70459. doi: 10.1371/journal.pone.0070459 (PMC3734283; doi:10.1371/journal.pone.0070459)
Supplement: Figure S2 — (PDF) [file pone.0070459.s002.pdf]

Benest et al., Supplementary Figure S2

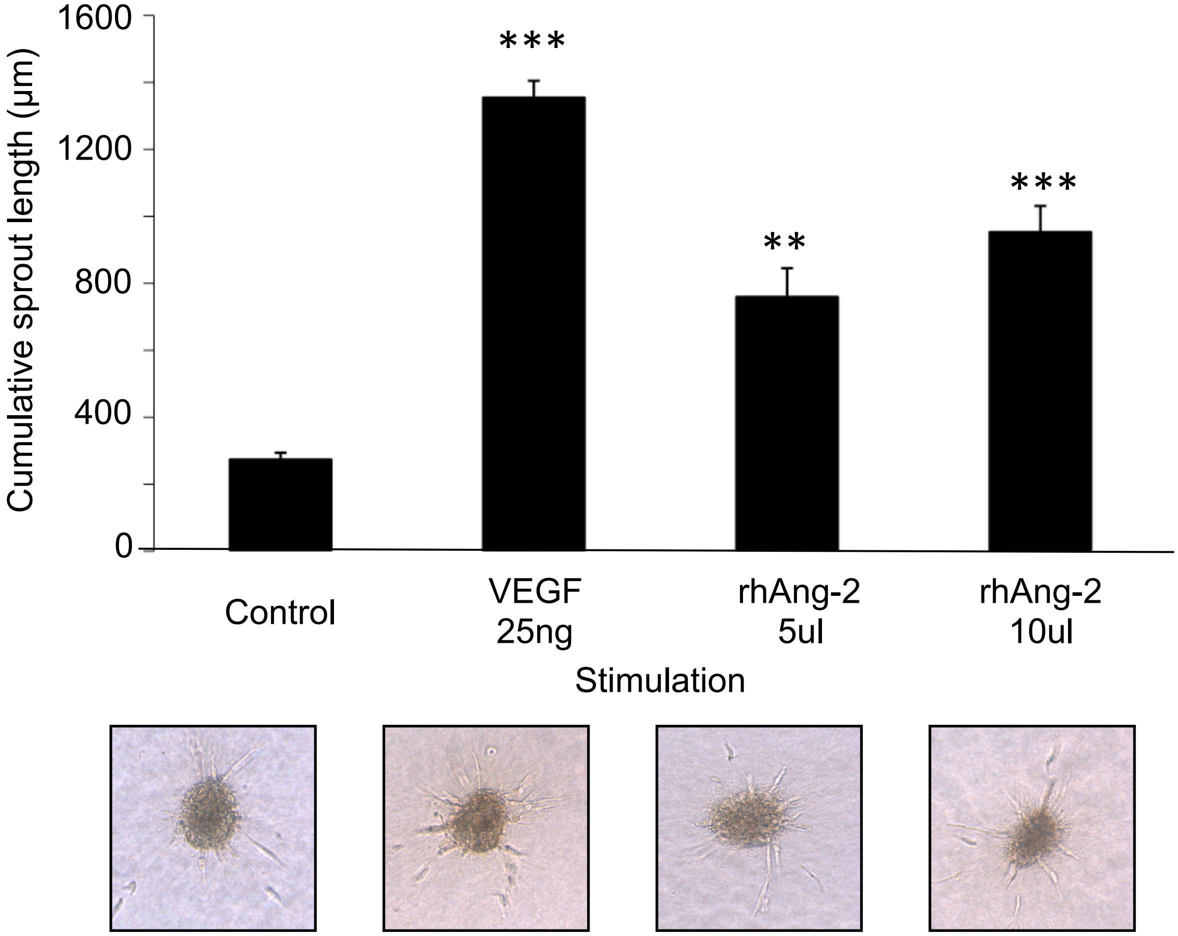

Supplementary Figure S2: Spheroid-based endothelial sprouting angiogenesis assay demonstrating the bioactivity of baculovirus-produced Ang-2. VEGF served as positive control. ** p<0.01, ***p<0.001 compared to control.
