# Supplementary material for: Angiopoietin-2 Is Critical for Cytokine-Induced Vascular Leakage
Source: PLoS One. 2013 Aug 5;8(8):e70459. doi: 10.1371/journal.pone.0070459 (PMC3734283; doi:10.1371/journal.pone.0070459)
Supplement: Figure S3 — (PDF) [file pone.0070459.s003.pdf]

Benest et al., Supplementary Figure S3

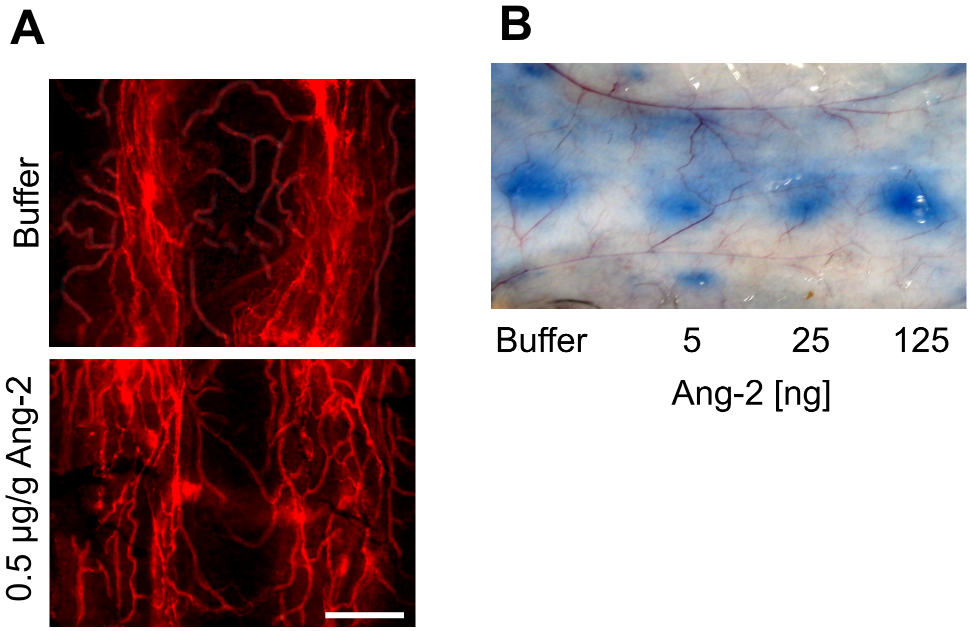

Supplementary Figure S3: Representative images of CD31-stained tracheal vasculatures (**A**) and Miles' Assay (**B**) following rhAng-2 administration. Scale bar 100 µm.
